# Epidemiological Investigation of Porcine Pseudorabies Virus in Hebei Province, China, 2017–2018

**DOI:** 10.3389/fvets.2022.930871

**Published:** 2022-06-24

**Authors:** Cheng Zhang, Huan Cui, Wuchao Zhang, Lijia Meng, Ligong Chen, Zhongyi Wang, Kui Zhao, Zhaoliang Chen, Sina Qiao, Juxiang Liu, Zhendong Guo, Shishan Dong

**Affiliations:** ^1^College of Veterinary Medicine, Hebei Agricultural University, Baoding, China; ^2^Changchun Veterinary Research Institute, Chinese Academy of Agriculture Sciences, Changchun, China; ^3^College of Animal Medicine, Jilin University, Changchun, China; ^4^Beijing Institute of Biotechnology, Beijing, China

**Keywords:** pseudorabies virus, epidemiological analysis, virus isolation, phylogenetic analysis, serological investigation

## Abstract

Pseudorabies (PR) is a serious disease affecting the pig industry in China, and it is very important to understand the epidemiology of pseudorabies virus (PRV). In the present study, 693 clinical samples were collected from Bartha-K61 vaccinated pigs with symptoms of suspected PRV infection between January 2017 and December 2018. All cases were referred for full clinical autopsy with detailed examination of histopathological examination, virus isolation and genetic evolution analysis of the PRV glycoprotein E (*gE*) gene. In addition, PRV *gE* antibodies in 3,449 serum samples were detected by the enzyme-linked immunosorbent assay (ELISA). The clinical data revealed that abortion and stillbirth are the most frequent appearances in pregnant sows of those cases. Histopathological examination exhibited a variety of pathological lesions, such as lobar pneumonia, hepatitis, lymphadenitis, nephritis, and typical nonsuppurative encephalitis. A total of 248 cases tested positive for the PRV gE gene. 11 PRV variants were isolated and confirmed by *gE* gene sequencing and phylogenetic analysis. These strains had 97.1%-100.0% nucleotide homology with the PRV reference strains. Notably, the isolated strains were highly homologous and clustered in the same branch as HSD-1/2019, which caused human acute encephalitis. Serological tests showed that the positive rate of PRV *gE* antibody in the 3449 serum samples collected from the Hebei Province was 46.27%. In conclusion, PRV variant strains Are high prevalence in the Hebei Province, which not only causes huge economic losses to the breeding industry but also potentially poses a threat to public health.

## Introduction

Pseudorabies (PR) is an acute infectious disease caused by pseudorabies virus (PRV) in many domestic animals and wild animals (1). It has a wide range of hosts and can infect mammals such as deer, bears, wolves, birds and humans (2, 3). Pigs are considered as the main and intermediate host of the PRV (4). The PRV mainly damages the reproductive system, respiratory system and nervous system of pigs in different ages. PRV is characterized by reproductive dysfunction of pregnant sows, neurological symptoms and high mortality of suckling piglets, and it is one of the important diseases harmful to the healthy development of the pig industry (1, 5–7).

The glycoprotein E (*gE*) gene is the main virulence gene of PRV, and the protein encoded by the *gE* gene plays an important role in mediating the fusion of virus entry, the release of virus particles, and the neurotrophic activity of viruses (8). PRV with *gE* gene deletion maintained immunogenicity but significantly reduced virulence (9). Before 2011, PR was largely controlled by the widespread application of the *gE* deletion vaccine in China (10). However, since 2012, there have been increasing reports of PR occurring in pigs vaccinated with the Bartha-K61 vaccine due to the emergence of variant strains of PRV (11–13). According to previous reports, the widely used PRV Bartha-K61 strain was shown to be incapable of providing complete protection against this new PRV variant (14). Despite great efforts to eradicate PRV in China, PR remains a serious threat to the Chinese pig industry (10, 15). During 2012–2021, the emergence of mutant PRV strains was reported in most provinces of China, resulting in the death of many pigs and huge economic losses (16). Since 2017, China has reported at least 14 cases of human infection with PRV, and a study in the cerebrospinal fluid of patients with an isolated first anthropogenic PRV strain HSD-1/2019 (17–23).

As an important area for pig breeding in China, Hebei Province has not been reported on the epidemiology of PRV before our study, so the investigation of the epidemiology of PRV in Hebei Province is of great significance. In this study, a total of 693 suspected clinical cases of PRV infection and 3,449 pig serum samples were collected from Hebei Province during the 24-month period from January 2017 to December 2018. Pathological examination, histological observation, virus isolation and identification, genetic evolution analysis and antibody level analysis were carried out. We found that the PRV variant strain is still prevalent in Hebei and poses a threat to the pig industry and public health.

## Materials and Methods

### Clinical Cases and Sample Collection

A total of 693 clinical cases of suspected PRV infection in 299 pig farms were collected during 2017–2018, which covered almost all cities of Hebei Province (Table 1). All pigs in this study had been inoculated with live PRV vaccine (Bartha-K61 strain). All examination and sample collection of the pigs were conducted in a biosafety laboratory. Corresponding tissue samples comprising brain, lung, liver, lymph node and kidney were collected from each pig. Samples were split into two groups, one of which was used to detect pathogens by polymerase chain reaction (PCR) or reverse transcription-polymerase chain reaction (RT–PCR), and another spot sample was fixed with 10% neutral formalin and used for hematoxylin-eosin (HE) staining and immunohistochemical (IHC) staining.

**Table 1:** Information on the clinical cases in this study.

**Origin city**	**Numbers**	**Clinical symptoms**				
		**High temperature**	**Abortion**	**Stillbirth**	**Neurological disorders**	**Respiratory problems**
Shijiazhuang	167	14	40	52	35	26
Baoding	104	10	20	25	26	23
Xingtai	78	5	19	24	20	10
Hengshui	37	0	12	19	0	6
Langfang	59	9	16	25	3	6
Zhangjiakou	74	3	20	35	16	0
Cangzhou	96	0	30	27	21	18
Tangshan	52	6	5	24	8	9
Handan	26	4	15	7	0	0
Total numbers	693	51	177	238	129	98

### Histopathologic Examination

Histopathological testing was performed using samples that were positive for PRV by PCR. The samples fixed in 10% neutral formalin were embedded in paraffin, and the tissue samples were cut into 4 mm thick sections. Hematoxylin and Eosin Staining Kit (C0105M, Beyotime, China) was used for HE staining and two-step anti-mouse IgG-HRP immunohistochemistry Kit (SV0001, BOSTER, China) was used for IHC staining. HE and IHC staining was performed following the manufacturer's instructions. The primary antibody used in IHC staining was mouse anti-PRV-gE mAb (LD-DW-Z0022, LV DU, China), diluted 1:1000.

### Virus Detection

DNA and RNA were extracted from collected tissue samples using the EasyPure^®^ Viral DNA/RNA Kit (ER201-01, TRANS, China) according to the manufacturer's instructions. RNA was converted into cDNA by the PrimeScript™ RT Reagent Kit with gDNA Eraser (Takara, Dalian, China). Tissue samples were analyzed for the presence of porcine circovirus type 2 (PCV2), porcine circovirus type 3 (PCV3), classical swine fever virus (CSFV), porcine reproductive and respiratory syndrome virus (PRRSV), or porcine epidemic diarrhea virus (PEDV) by PCR or RT–PCR as previously reported (24). The primers used in this study are listed in Supplementary Table 1.

### Isolation and Identification of Virus

All PRV positive samples were homogenized in Dulbecco's modified Eagle's medium (DMEM) (Gibco, Grand Island, NY, USA). The sample was then centrifuged at 8,000 × g for 15 min at 4°C, and the supernatants were filtered through 0.22 μm membrane filters (Millipore, Billerica, MA, USA). The monolayer porcine kidney cell line PK-15 (CCL-33, ATCC, USA) was incubated with filtered supernatant for 1.5 h. DMEM was supplemented with 10% fetal bovine serum (Gibco, Grand Island, NY, USA), 100 μg/mL streptomycin, and 100 U/mL penicillin. The inoculated PK-15 cells were placed in a 5% CO_2_ incubator at 37°C. Cells were observed daily for cytopathic effect (CPE). When the CPE reached 80%, the cells were harvested, frozen and thawed three times. The virus was further purified by plaque assay, and the isolated virus was identified by RT–PCR or PCR.

### Sequence and Genetic Evolution Analysis

The extracted PRV DNA was amplified by PCR according to previous studies to obtain the full length of the *gE* gene (25). The full-length *gE* gene primers are shown in Supplementary Table 1. Sequencing was performed by Comate Biotech Company (Jilin, China). The sequences obtained were submitted to the GenBank database (Supplementary Table 2). Reference sequences of *gE* genes were downloaded from NCBI GenBank (Supplementary Table 2), and the downloaded sequences were aligned and compared with the strains in this study using Cluster W. Phylogenetic analysis was performed using MEGA7.0.21 software (Sinauer Associates, Inc., Sunderland, MA, USA) based on the maximum likelihood (ML) with a bootstrap value of 1,000.

### Serologic Assays

A total of 3,449 serum samples were collected from 2017 to 2018, covering almost all of Hebei Province, and the collection locations, growth stages and scales of farms of serum samples were recorded in detail (**Tables 3**, **4**). Commercial ELISA kits (IDEXX Laboratories, Westbrook, ME, USA) were used to detect PRV *gE* antibody levels in serum samples to distinguish vaccine strains from wild-type virulent PRV strains, according to the manufacturer's instructions.

## Results

### Clinical Symptoms

**Figure 1:**
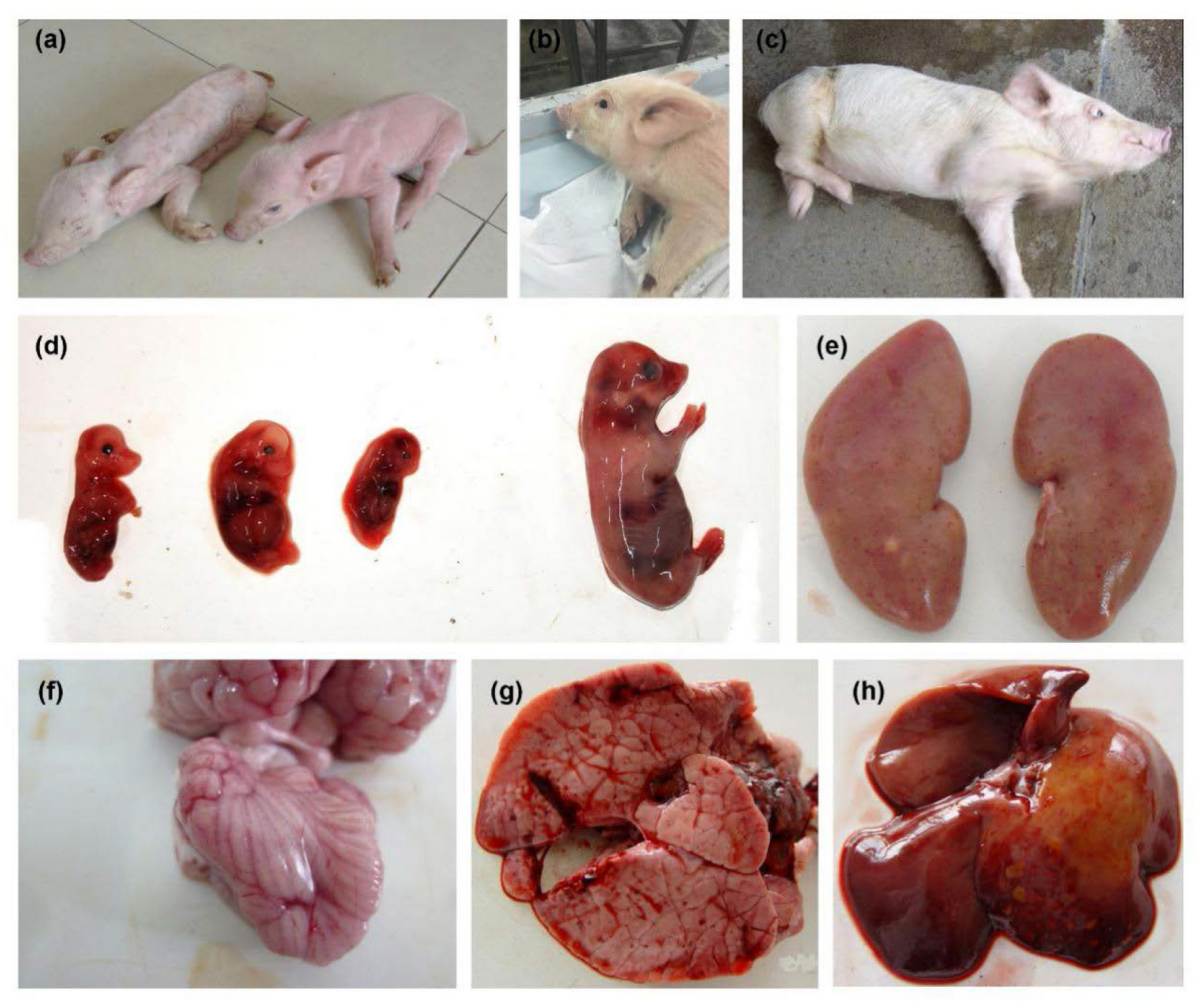
Clinical symptoms observed in pseudorabies virus-infected pigs. **(A)** Giving birth to weak piggies. **(B)** Foaming at the mouth. **(C)** Severe neurological disorders. **(D)** Aborted fetus. **(E)** Hemorrhagic spot on the renal cortex. **(F)** Cerebral hemorrhage and congested meninges. **(G)** Pulmonary hemorrhage. **(H)** Liver with multiple small focal areas of necrosis.

The body temperature of the PRV-infected sows reached 40–41°C, accompanied by phenomena such as giving birth to weak pigs (Figure 1A). The newborn piglets were weak in their capacity to suck the breast. Soon after, empty chewing and molars were seen, leading to a large amount of foam liquid flowing from the corner of the mouth (Figure 1B). Flush conjunctiva, turbid cornea, eyelid edema, eye fixation and glazed eyes were observed. Later, dyskinesia of the posterior limbs and a shaking body were found (Figure 1C). Abortion was also a common symptom (Figure 1D). Hemorrhagic spots were observed on the renal cortex (Figure 1E). Cerebral hemorrhage and congested meninges were visible (Figure 1F). The congested lung was swollen with focal white necrosis, and severe pulmonary hemorrhage was also observed (Figure 1G). Multiple small focal areas of necrosis were observed in the liver (Figure 1H). According to Table 1, abortion and stillbirth occur most frequently in the clinical manifestations of pigs suspected of PRV infection. Neurological disorders and respiratory problems were moderate, and high temperature was the least common.

### Observation of Histopathological Changes

**Figure 2:**
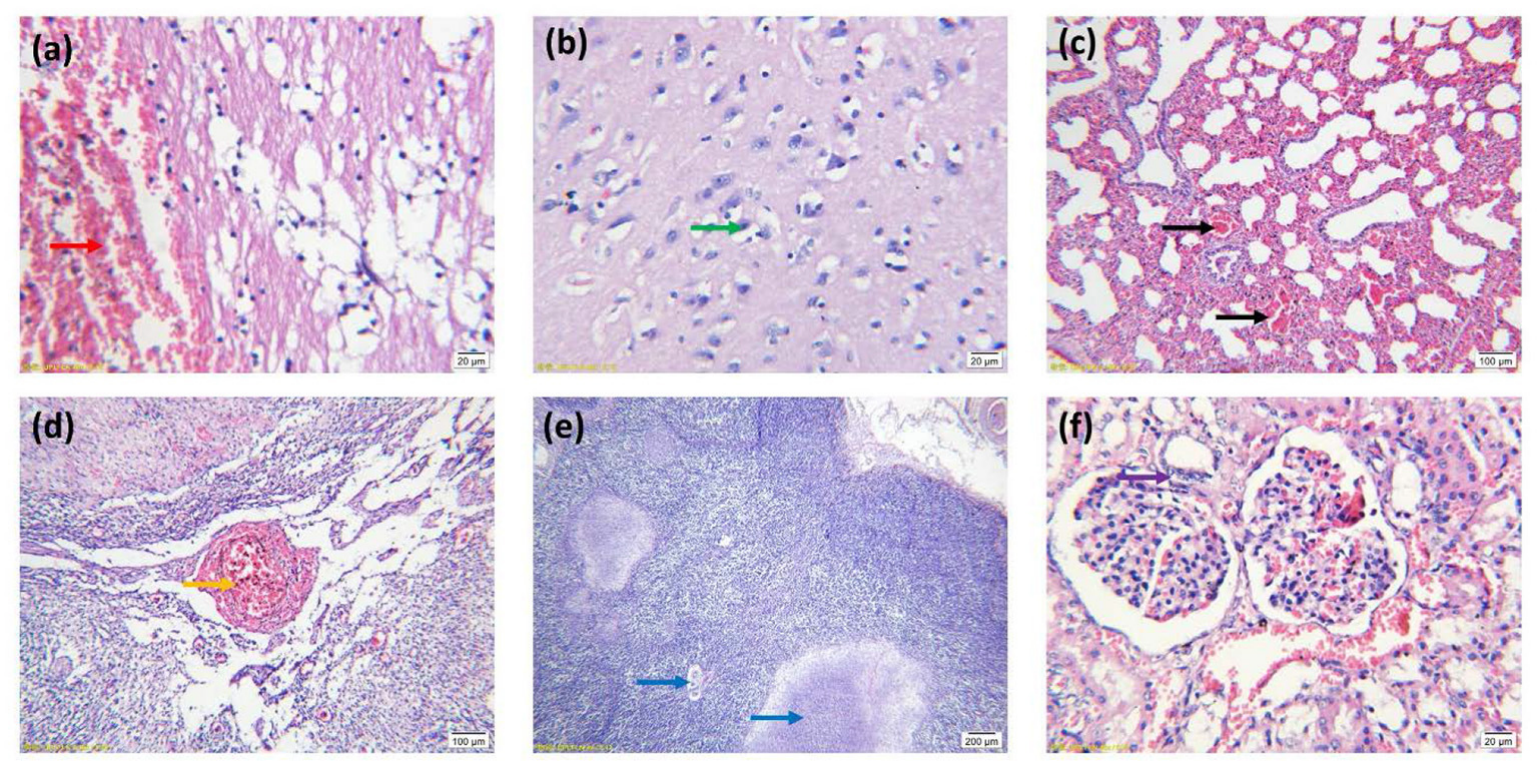
Histopathological features of tissues stained with hematoxylin and eosin. **(A)** Focal hemorrhage of the brain (arrow red). **(B)** Necrosis and neuronophagia of neurocytes (arrow green). **(C)** Lung congestion (arrow black). **(D)** Congestion and hemorrhage of lymph nodes (arrow orange). **(E)** Necrosis of lymphoid follicles (arrow blue). **(F)** Degeneration and necrosis of renal tubular epithelial cells (arrow purple). **(A,B,F)** Images were obtained at 100 × magnification; **(C,D)** Images were obtained at 20 × magnification; **(E)** Images were obtained at 10 × magnification.

The results of pathological observations are shown in Figure 2. The brain had multiple symptoms, including focal hemorrhage, focal vacuolation of brain parenchyma, a large amount of lymphocyte infiltration, degeneration, necrosis and neuronophagia of neurocytes, hematoxylin and congestion and formation of perivascular cuffing, forming typical nonsuppurative encephalitis. For the lung, capillaries in the alveolar walls were congested, and alveolar walls were thickened. A large number of erythrocytes, necrotic and exfoliated epithelial cells, lymphocytes and incarnadine inflammatory protein and tissue liquid were observed in the alveolar space. The smooth muscle layer of the vascular walls was necrotic, thickened and congested. Partial epithelial cells of the bronchial mucosa were necrotic and had a great number of inflammatory cells, which were mainly lymphocytes as well as exfoliated epithelial cells. Loosening and edema were obvious in the mesenchyme surrounding the bronchiole, and there was a bulk of lymphocytes and erythrocytes within. Obvious lobar pneumonia was formed. The liver had multiple symptoms, including hepatic sinus expansion and congestion, hepatic steatosis and necrosis, and a large amount of lymphocyte infiltration in the hepatic lobule indicated degenerative hepatitis. The lymph nodes had multiple symptoms including congestion and hemorrhage of lymph nodes, decreased lymphoid follicles and necrotic and disintegrated lymphocytes. Degenerated necrosis and hemorrhage of the vascular wall were observed. The tonsilla had multiple symptoms including congestion and hemorrhage, degeneration and necrosis of epithelial cells. Necrosis of lymphocytes in lymphoid nodules. The kidney had multiple symptoms including congestion and hemorrhage, degeneration and necrosis of renal tubular epithelial cells. A large amount of incarnadine protein and exfoliated epithelial cells were observed in the cavity.

**Figure 3:**
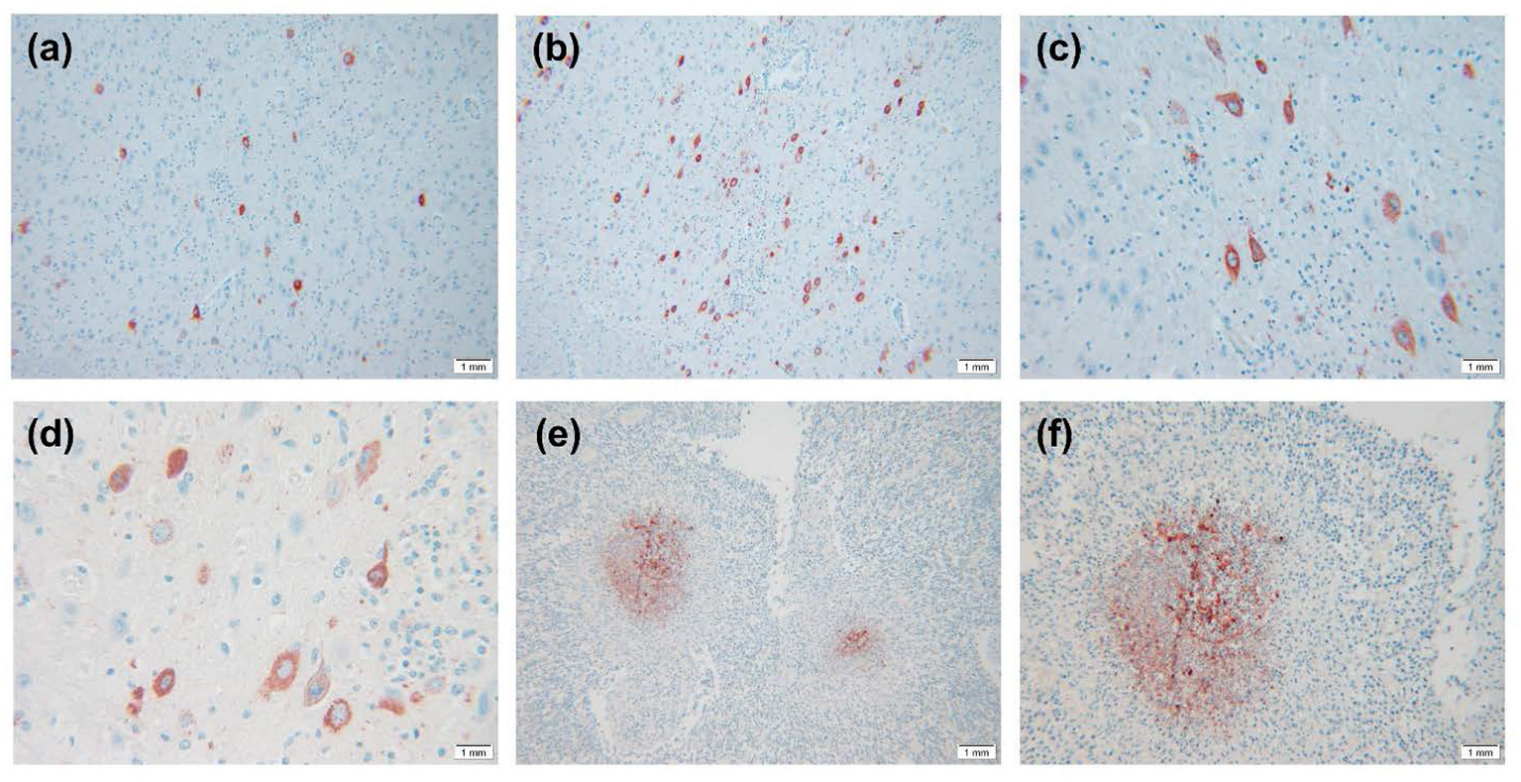
IHC results of PRV-infected pig tissue. **(A–D)** Virus-positive particles were widely found in the brain, and virus-positive particles mainly appeared in the cytoplasm and axons of neurons in the brain. **(E,F)** There were a large number of virus-positive particles in the cortex of lymph nodes, and the virus-positive particles mainly existed in lymphocytes and macrophages. Images were obtained at 400 × magnification.

The IHC results are shown in Figure 3. Virus-positive particles were widely found in the brain, and they mainly appeared in the cytoplasm and axon of neurons (Figures 3A–D). The cortical area of the lymph node contains many positive particles, indicating the presence of PRV in the cortical area, and the virus-positive particles mainly exist in lymphocytes and macrophages (Figures 3E,F).

### *gE* Genes Positive Rate

During the 24 months from January 2017 to December 2018, 693 suspected PRV infection cases from Hebei Province were collected. Among the suspected PRV infection cases, the *gE* gene was detected in 248 (35.78%) of 693 suspected cases of PRV infection, in which PCV2, PCV3, PRRSV and CSFV were negative.

### Evolutionary Genetic Analysis

**Figure 4:**
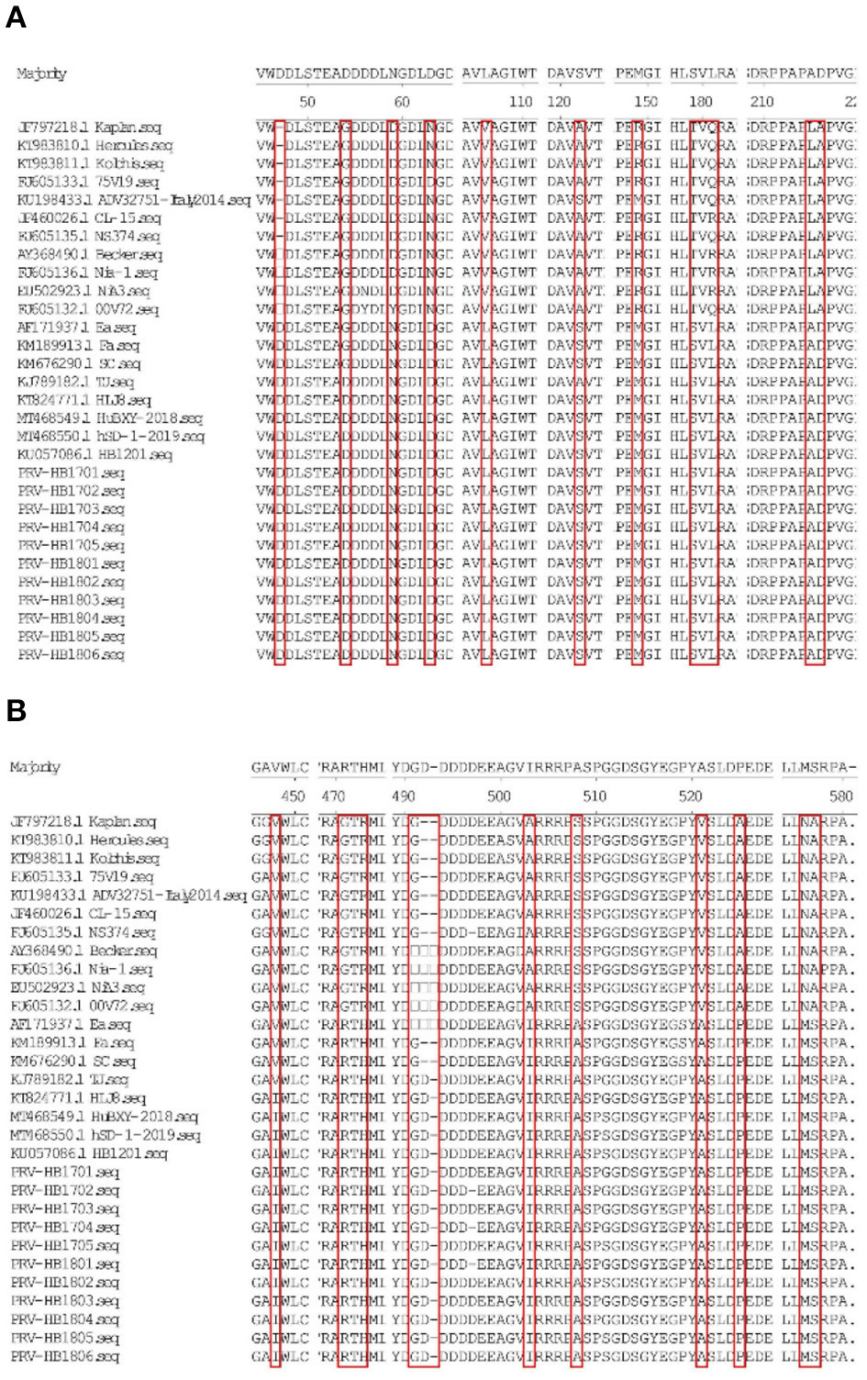
**(A,B)** Show the amino acid (aa) changes of the 11 PRV isolates compared to the reference strains.

**Figure 5:**
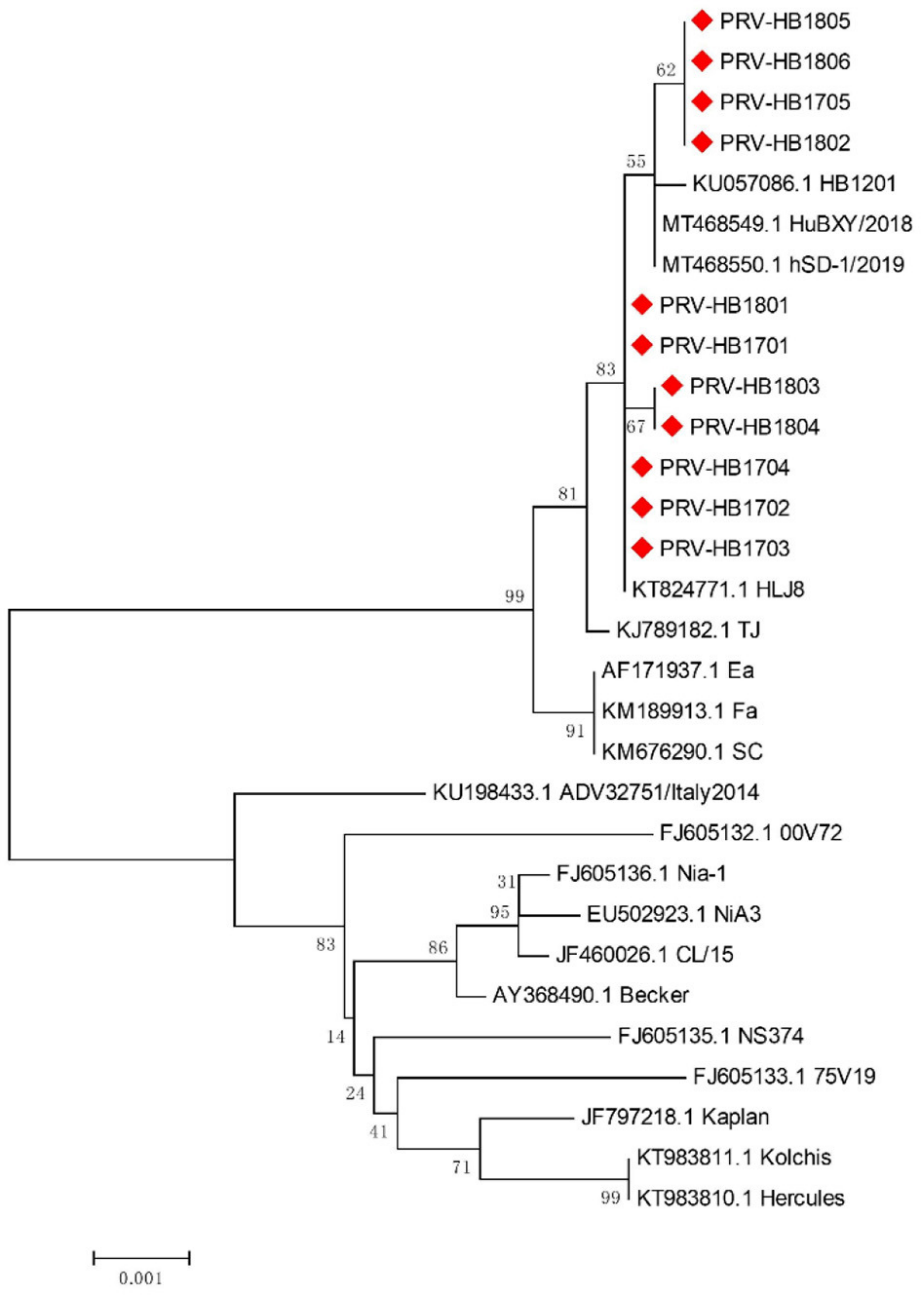
Phylogenetic analysis and comparison based on pseudorabies virus (PRV) glycoprotein E (*gE*) nucleotide sequences. Red diamonds, 11 PRV isolates from Hebei Province collected from 2017 to 2018.

11 strains with different gE gene sequences were obtained after 248 PRV-gE gene repeats were deleted. Analysis of the 11 sequences showed that the nucleotide homology and amino acid homology were 97.1–100.0% and 95.0–100.0%, respectively, with the reference strain (Table 2), which were highly homologous with HSD-1/2019 causing human acute encephalitis. Compared with the reference strains, the changes in gE genes in this study are shown in Figure 4, which are highly consistent with the epidemic strains and human infection strains in recent years. Phylogenetic analysis of the gE gene showed that PRV isolates from China were located on a separate phylogenetic clade from isolates from other countries (Figure 5). In this branch, the gE genes of the 11 strains in this study were adjacent to PRV variants TJ, HLJ8 and HB1201, and closely related to HSD-1/2019. The gE genes of the 11 strains in this study were far different from those of Fa, Ea and SC. These results indicate a close phylogenetic relationship between the isolates in this study and PRV variants in China, including HSD-1/2019, which causes human acute encephalitis.

**Table 2:** Nucleotide and amino acid identities for glycoprotein E (*gE*) gene between 11 gE genes and that of 19 representative PRV isolates (%).

**Identities**	**Nia-1**	**NS374**	**CL-15**	**75V19**	**00V72**	**Becker**	**NiA3**	**Hercules**	**Kolchis**	**Kaplan**	**ADV32751-Italy2014**
Origin	Ireland	Belgium	Argentina	Belgium	Belgium	USA	Spain	Greece	Greece	Hungary	Italy
Isolate time	1962	1971	1971	1975	2000	2003	2008	2010	2010	2011	2014
Accession no.	FJ605136	FJ605135	JF460026	FJ605133	FJ605132	AY368490	EU502923	KT983810	KT983811	JF797218	KU198433
Nt	97.2-97.4	97.6-97.7	97.7-97.9	97.5-97.6	97.1-97.2	97.9-98.0	97.7-97.8	97.6-97.8	97.6-97.8	97.7-97.8	97.9-98.0
AA	95.3-95.5	95.5-95.7	95.8-96.0	96.5-96.7	95.0-95.2	95.8-96.0	95.7-95.8	96.0-96.2	96.0-96.2	96.0-96.2	95.8-96.0
**Identities**	**SC**	**Ea**	**Fa**	**HB1201**	**HLJ8**	**TJ**	**HuBXY/2018**	**hSD-1/2019**
Origin	China	China	China	China	China	China	China	China
Isolate time	1986	1999	2012	2012	2013	2014	2018	2019
Accession no.	KM676290	AF171937	KM189913	KU057086	KT824771	KJ789182	MT468549	MT468550
Nt	99.6-99.7	99.6-99.7	99.6-99.7	99.6-99.9	99.9-100.0	99.8-99.9	99.9-99.9	99.9-99.9
AA	99.1-99.3	99.1-99.3	99.1-99.3	99.8-100.0	99.8-100.0	99.5-99.7	99.8-100.0	99.8-100.0

*Nt, nucleotide; AA, amino acid*.

### Serological Investigation of Serum Samples

A total of 3449 serum samples were collected from January 2017 to December 2018 in Hebei Province, of which 1596 were positive for gE-specific antibodies, and the overall seropositivity rate (SRP) for the study was 46.27%. Tables 3, 4 show the SPR of different regions, production stages and breeding scales. According to the data in Table 4, Shijiazhuang city has the highest SPR (59.6%), and Baoding city has the lowest SPR (30.8%). The SPR of other cities ordered from high to low is Tangshan city (56.9%), Zhangjiakou city (53.3%), Langfang city (52.9%), Handan city (51.7%), Hengshui city (49.1%), Cangzhou city (38.3%), and Xingtai city (37.1%). Meanwhile, the data in Table 3 also show the SPR of different stages, and the SPR of growing finishing pigs was the highest (59.7%). They were followed by nursery pigs (54.9%), suckling piglets (48.8%), multiparous sows (46.8%), boars (24.5%) and gilts (19.7%). As shown in Table 4, the SPR of pig farms with basal sows≥3,000 pigs is the lowest (27.4%), and the SPR of basal sows of 200–500 pigs is the highest (55.7%). The SPR of pig farms with 500–1,000 basal sows was 51.6% and that of pig farms with 1000–3000 basal sows was 48.7%.

**Table 3:** Information on the serum samples from 2017 to 2018.

**Origin city**	**Serum numbers**	**Positive** **number**	**SPR (%)**	**Herds**					
				**Boars**	**Gilts**	**Multiparous** **sows**	**Suckling piglets**	**Nursery** **pigs**	**Growing- finishing pigs**
Shijiazhuang	990	590	59.6	37	68	494	110	192	89
Baoding	1,097	338	30.8	24	54	707	102	142	68
Xingtai	213	79	37.1	11	27	54	54	46	21
Hengshui	57	28	49.1	10	10	17	3	13	4
Langfang	87	46	52.9	18	21	13	5	17	13
Zhangjiakou	629	335	53.3	20	45	357	80	47	80
Cangzhou	149	57	38.3	13	26	27	27	48	8
Tangshan	109	62	56.9	18	23	13	27	14	14
Handan	118	61	51.7	4	10	46	20	22	16
Total number	3,449	-	-	155	284	1,728	428	541	313
Positive number	1,596	-	-	38	56	809	209	297	187
SPR (%)	46.27	-	-	24.5	19.7	46.8	48.8	54.9	59.7

**Table 4:** Serum sample information from farms of different sizes.

**Number of basal sows**	**Serum numbers**	**Positive number**	**SPR (%)**	**Sample percentage**
200–500	978	545	55.7%	28.4%
500–1,000	892	460	51.6%	25.9%
1,000–3,000	743	362	48.7%	21.5%
≥3,000	836	229	27.4%	24.2%

## Discussion

PR has become a recognized infectious disease, which causes huge economic losses to the breeding industry and poses a threat to public health security. Since 2017, China has reported at least 14 cases of human infection with PRV. Considering these cases of human PRV infection and the close relationship between pigs and humans, a large ammount of clinical cases and serum samples were collected from 2017 to 2018 in Hebei Province, China for the study.

It has been reported that pigs infected with the PRV variant strain showed severe clinical symptoms such as high fever, tonsil bleeding, lung swelling and necrosis (26). Among the pigs suspected of PRV infection, abortion and stillbirth have the most occurrence (27), which is consistent with the cases in our study. HE staining analysis showed typical pathological changes in the brain and lungs of PRV-infected pigs, including nonsuppurative encephalitis and lobular pneumonia, and immunohistochemical staining showed multiple positive staining signals in the brain, indicating that PRV had strong neurotropic characteristics. At the same time, lymph node hyperemia, hemorrhage, lymphatic follicle reduction, lymphocyte necrosis, disintegration and other pathological changes were also observed. There were many positive particles in the cortical region of lymph nodes, indicating the presence of PRV in the cortical region.

From 2011 to 2021, the positive rate of PRV *gE* gene nucleic acid in pigs in China was 11.5% (16), and the prevalence of PRV was closely related to region (25). In this study, from January 2017 to December 2018, we collected a total of 693 suspected cases of PR infection in Hebei Province, China, of which 248 (35.78%) were PRV *gE* gene positive. The results of our study are much higher than those of previous studies, which may be related to sample collection methods and geographical locations. Compared with other studies, the increase in the PRV *gE* gene-positive rate was closely related to the relatively backward breeding production level and lax production management in Hebei Province. The high positive rate of the *gE* gene in Hebei Province once again highlights the importance of continuous monitoring of PRV. In clinical cases, PRV is commonly coinfected with other viruses, such as PRRSV, CFSV, PCV2, PCV3, etc. (28). In this study, mixed infection of a variety of viruses also appeared, indicating that other diseases are prevalent in Hebei, which requires further research.

The *gE* gene of 11 PRV strains isolated in this study had high homology with PRV variant strains, including the human PRV strain HSD-1/2019. The PRV *gE* gene is closely associated with virulence (15). Alignment of amino acid sequences revealed that the same amino acid mutations as previously reported were found in *gE* compared to the reference strain (27). Mutations in the *gE* gene may affect the virulence of isolates, which requires further study. The *gE* genes of the 11 strains in this study were in the same clade as the PRV variants TJ, HLJ8 and HB1201 and were closely related to HSD-1/2019. Traditional PRV vaccines cannot provide sufficient cross-protection against PRV variant isolates (15). In fact, since 2012, PRV variant strains have been widely spread in China, and there have been increasing reports of human infection with PRV, so more attention should be given to PRV (16).

A recent study collected data from 2011 to 2020 and found that the seroprevalence of PRV *gE* in China was 29.87% (76,553/256,326) (16). The seroprevalence of PRV *gE* varies in different regions of China. In Henan Province, 30.14% (1,419/4,708) of the samples collected in 2018–2019 were *gE* seropositive (10), while in Heilongjiang Province, 16.3% (3,067/18,815) were *gE* seropositive from 2013 to 2018 (29). The positive rate of *gE* in Shandong Province from 2013 to 2016 was 57.8% (2,909/5,033) (27). In this study, the positive rate of *gE* antibody in serum samples was 46.27%, similar to that in Shandong Province but different from that in other provinces. The positive rate of serum *gE* was different among different cities in Hebei Province, but it remained at a high level in general. The reason for this situation may be that some pig farms did not test in advance when they introduced (or retained) breeding pigs, leading to the introduction of positive and toxic backup breeding pigs. Meanwhile, due to unreasonable immunization procedures, some pig farms were infected with wild viruses after the introduction of negative backup breeding pigs, leading to positive antibodies. In this situation, new strains of infection appeared. The decreased immune effect of the previous vaccine may have caused the increase in the positive rate in Hebei Province. Our data also showed that the growing-finishing pigs had the highest SPR, followed by the nursery pigs, suckling piglets, multiparous sows, boars and gilts. We found that with the increase in the weeks of age of commercial pigs, the positive rate showed an increasing trend, which also reflected the gradual increase in infection risk. The *gE* seropositivity of multiparous sows was 46.8% (809/1728), which is consistent with previous studies indicating that multiparous sows are at high risk of PRV infection (27). Piglets infected with PRV through vertical transmission can cause persistent and recurrent infection in pig herds. By comparing four farms of different sizes, it was found that the larger the breeding scale was, the lower the *gE* positive rate was. Large-scale pig farms in Hebei Province generally adopt a three-point or multipoint breeding mode (a pig farm is divided into different areas according to the function of different piggery, the different areas are relatively closed, and different week-old pigs are transported to the specified piggery by field transport vehicles). These two models reduce the chance of return from high-risk herds to breeding herds, which helps control the disease. In addition, large-scale pig farms pay more attention to biosafety, reducing the risk of infection. All the data showed that the seropositive rate of PRV variant strains was still high in Hebei Province, China, which posed a challenge to pig breeding in Hebei Province, China.

In this study, systematic investigation including clinical autopsy, histological examination, virus isolation, sequencing and phylogenetic analysis, and serological investigation showed that the PRV variant strain was still prevalent in Hebei Province, China, and the protective effect of current vaccines against the new strain was poor. With the widespread epidemic of PRV strains in pigs in Hebei Province, China, it can be predicted that this disease will still be one of the most important diseases affecting the healthy development of the pig industry in Hebei Province in the future. Our study did not last longer due to the impact of the African swine fever outbreak. In view of the increasing reports of PRV infection in humans after 2017. Future study should be carried out from the following aspects: Strengthen the feeding management of pigs by improving hardware and feeding nutritious feeds to avoid the prevalence of related “endogenous” diseases on farms. Establish high-level biosafety system and strengthen the biosafety awareness of farm staff to avoid the recurrence of PRV infection in human, and control the invasion of “exogenous diseases in pig farms” caused by seed introduction, traffic flow, human flow and logistics, etc. Define the idea of prevention and control of diseases in pig farms, make suitable immunization and health program to build up a protective shield for pig farms, and implement it firmly through production management. Regularly monitor the immunization and prevention and control of pig herd to minimize the danger of diseases. The prevention and control of PR should still focus on purifying pig farms. In conclusion, this study is helpful to analyze the epidemiological situation of PRV in Hebei Province and provide basic data for the prevention and control of PRV.

## Data Availability Statement

The datasets presented in this study can be found in online repositories. The names of the repository/repositories and accession number(s) can be found in the article/Supplementary Material.

## Ethics Statement

The animal study was reviewed and approved by Animal Ethics Committee of Hebei Agricultural University.

## Author Contributions

JL, SD, and ZG designed the project. CZ, HC, and WZ performed the experiments. LC, ZC, KZ, SQ, ZW, and LM analyzed the data. CZ, ZG, and HC drafted the manuscript. JL and SD critically revised the manuscript. All authors contributed to the article and approved the submitted version.

## Funding

This study was supported by the Key Research Projects in Hebei Province (20326622D and 18227517D) and Hebei Industrial Technology System (HBCT2018150210).

## Conflict of Interest

The authors declare that the research was conducted in the absence of any commercial or financial relationships that could be construed as a potential conflict of interest.

## Publisher's Note

All claims expressed in this article are solely those of the authors and do not necessarily represent those of their affiliated organizations, or those of the publisher, the editors and the reviewers. Any product that may be evaluated in this article, or claim that may be made by its manufacturer, is not guaranteed or endorsed by the publisher.
