# Supplementary material for: Epidemiological Investigation of Porcine Pseudorabies Virus in Hebei Province, China, 2017–2018
Source: Front Vet Sci. 2022 Jun 24;9:930871. doi: 10.3389/fvets.2022.930871 (PMC9263846; doi:10.3389/fvets.2022.930871)
Supplement: Supplementary file 1 [file Data_Sheet_1.DOCX]

**Supplementary Table 1 Primers used for PCR amplification in this study**

| **Primer** | **Sequence 5’-3’** | **Length** | **Purpose** | **Reference sequence** | |
| --- | --- | --- | --- | --- | --- |
| Detection-PRV-F | GACTACGCCGACTACTACG | 445 | Detection of PRV | | MK806387 |
| Detection-PRV-R | GAAGATGTCAGAGGGCGAG |  |  |  |  |
| Detection-PCV2-F | CATATGAAATAAATTACTGAG | 762 | Detection of PCV2 | | AY321991 |
| Detection-PCV2-R | CAGCGCACTTCTTTCGTTTTGA |  |  |  |  |
| Detection-PCV3-F | TACTTAGAGAACGGACTTGTAACG | 648 | Detection of PCV3 | | MN075133 |
| Detection-PCV3-R | AAATGAGACACAGAGCTATATTCA |  |  |  |  |
| Detection-CSFV-F | AACATGGATGGTGTAACTG | 320 | Detection of CSFV | | AY805221 |
| Detection-CSFV-R | TCTCTATAGTGTTGGTCATTCC |  |  |  |  |
| Detection-PRRSV-F | GAGTTTCAGCGGAACAATG | 432 | Detection of PRRSV | | KC527830 |
| Detection-PRRSV-R | CCGTTGACCGTAGTGGAG |  |  |  |  |
| PRV-*gE*-F | ATGCGGCCCTTTCTGCTGC | 1740 | Sequencing | | MK806387 |
| PRV-*gE*-R | TTAAGCGGGGCGGGACATCA |  |  |  |  |

**Supplementary Table 2 Basic information of PRV *gE* gene from 36 isolate and reference strains**

| **Accession number** | **Isolate** | **Country** | **Year of isolation** |
| --- | --- | --- | --- |
| FJ605136 | Nia-1 | Ireland | 1962 |
| FJ605135 | NS374 | Belgium | 1971 |
| JF460026 | CL-15 | Argentina | 1971 |
| FJ605133 | 75V19 | Belgium | 1975 |
| FJ605132 | 00V72 | Belgium | 2000 |
| AY368490 | Becker | USA | 2003 |
| EU502923 | NiA3 | Spain | 2008 |
| KT983810 | Hercules | Greece | 2010 |
| KT983811 | Kolchis | Greece | 2010 |
| JF797218 | Kaplan | Hungary | 2011 |
| KU198433 | ADV32751-Italy2014 | Italy | 2014 |
| KM676290 | SC | China | 1986 |
| AF171937 | Ea | China | 1999 |
| KM189913 | Fa | China | 2012 |
| KU057086 | HB1201 | China | 2012 |
| KT824771 | HLJ8 | China | 2013 |
| KJ789182 | TJ | China | 2014 |
| MT468549 | HuBXY/2018 | China | 2018 |
| MT468550 | hSD-1/2019 | China | 2019 |
| ON324025 | PRV-HB1701 | China | 2017 |
| ON324029 | PRV-HB1705 | China | 2017 |
| ON324026 | PRV-HB1702 | China | 2017 |
| ON324028 | PRV-HB1704 | China | 2017 |
| ON324027 | PRV-HB1703 | China | 2017 |
| ON324032 | PRV-HB1803 | China | 2018 |
| ON324030 | PRV-HB1801 | China | 2018 |
| ON324031 | PRV- HB1802 | China | 2018 |
| ON324034 | PRV-HB1805 | China | 2018 |
| ON324035 | PRV-HB1806 | China | 2018 |
| ON324033 | PRV-HB1804 | China | 2018 |
